# PPARβ Interprets a Chromatin Signature of Pluripotency to Promote Embryonic Differentiation at Gastrulation

**DOI:** 10.1371/journal.pone.0083300

**Published:** 2013-12-18

**Authors:** Nicolas Rotman, Nicolas Guex, Erwan Gouranton, Walter Wahli

**Affiliations:** 1 Center for Integrative Genomics, National Research Center “Frontiers in Genetics”, University of Lausanne, Lausanne, Switzerland; 2 Vital-IT group, Swiss Institute of Bioinformatics, Lausanne, Switzerland; 3 Lee Kong Chian School of Medicine, Nanyang Technological University, Singapore, Singapore; Michigan State University, United States of America

## Abstract

Epigenetic post-transcriptional modifications of histone tails are thought to help in coordinating gene expression during development. An epigenetic signature is set in pluripotent cells and interpreted later at the onset of differentiation. In pluripotent cells, epigenetic marks normally associated with active genes (H3K4me3) and with silent genes (H3K27me3) atypically co-occupy chromatin regions surrounding the promoters of important developmental genes. However, it is unclear how these epigenetic marks are recognized when cell differentiation starts and what precise role they play. Here, we report the essential role of the nuclear receptor peroxisome proliferator-activated receptor β (PPARβ, NR1C2) in *Xenopus laevis* early development. By combining loss-of-function approaches, large throughput transcript expression analysis by the mean of RNA-seq and intensive chromatin immunoprecipitation experiments, we unveil an important cooperation between epigenetic marks and PPARβ. During *Xenopus laevis* gastrulation PPARβ recognizes H3K27me3 marks that have been deposited earlier at the pluripotent stage to activate early differentiation genes. Thus, PPARβis the first identified transcription factor that interprets an epigenetic signature of pluripotency, in vivo, during embryonic development. This work paves the way for a better mechanistic understanding of how the activation of hundreds of genes is coordinated during early development.

## Introduction

How a single egg cell divides and invariably produces all sorts of differentiated cells that form the adult organism is the foundational question of developmental biology. Because all the cells of the organism share the same genome inherited from the zygote, different epigenetic landscapes are the main distinctive genomic feature of differentiated cells. This points to the important role of heritable epigenetic marks such as DNA methylation, histone H3 tri-methylation on lysine 4 (H3K4me3) or on lysine 27 (H3K27me3) in the process of differentiation [1], [2]. Epigenetic marks not only contribute to the progression of cell differentiation, they also help in coordinating the switch from pluripotency to early differentiation. Indeed, an epigenetic signature is set in pluripotent cells before the onset of differentiation. In cultured embryonic stem cells (ESCs) and pluripotent stem cells, and the developing fish embryo, ‘bivalent’ genes bear opposing epigenetic marks in the vicinity of their promoter region: the activating H3K4me3 and the repressive H3K27me3 [3], [4]. These bivalent genes mostly encode important developmental regulators [3], [5], [6], [7], [8], and many are synchronously induced during cell differentiation, concomitantly losing their repressive H3K27me3 mark [5], [6], [7], [9]. This process is thus thought to facilitate a coordinated wave of gene expression by identifying the few hundred genes that are important for the very early differentiation in the total repertoire of more than 20,000.

However, the relation between gene expression at the onset of differentiation and bivalency is complex: many bivalent genes are synchronously activated [3], [5] while some others become repressed [10]. Therefore, it is likely that other factors should be taken into consideration, and in particular the transcription factors that should interpret this epigenetic signature at the time of differentiation. However, if common signal transducers are likely implicated in H3K27me3 loss at bivalent genes during differentiation in ESC, how specific genes are recognized is unclear and in vivo knowledge is limited [11], [12], [13].

Here we report that the nuclear hormone receptor peroxisome proliferator-activated receptor β (PPARβ, NR1C2) can interpret an epigenetic signature of pluripotency during *Xenopus laevis* gastrulation.

PPARβ is a ligand-activated transcription factor important for cell differentiation in the adult [14] and in the placenta [15], but its role in the embryo has not been studied properly. We show that in *Xenopus laevis*, PPARβ is essential for neural and muscle differentiation as early as in gastrulation when a massive change in transcript level occurs. By using genomic (RNA-seq) and bioinformatics approaches we propose that PPARβ preferentially activates bivalent genes at gastrulation. This hypothesis is supported by direct examination of developing *Xenopus laevis* embryos using chromatin immunoprecipitation (ChIP). Most importantly, pharmacologic manipulation of H3K27me3 levels in the embryo indicates that it is this mark that triggers gene activation by PPARβ. Our work represents an important step towards a better understanding of the role of epigenetic marks in the switch from pluripotent to differentiated state.

## Materials and Methods

### Animal care and housing

Animal care and handling procedures were approved by the Commission de Surveillance de l’Expérimentation Animale of the Canton de Vaud, Switzerland.

### In vitro fertilization, embryo microinjections, and drug treatments

*X. laevis* oocyte collection, fertilization, and de-jellying were performed according to standard procedures [16]. Unless otherwise indicated, injections were done at the 2-cell stage, with a single injection in each of the two cells. 3-deazaneplanocin A (DZNep) (Cayman) treatment: *X. laevis* embryos were injected with Co or PPARβ MO (morpholino) and allowed to develop until stage 5 when DZNep or DMSO was added to the water. Embryos were collected at stage 10.5 for RT-qPCR or ChIP with H3K27me3 antibody.

### RNA–seq

The paired-end tags with no more than 5 N in each pair were mapped onto 10,691 distinct RefSeq *X. laevis* mRNA with fetchGWI, which is part of the Tagger software suite [17], using up to one mismatch per tag. Paired-end tags matching a unique mRNA with a separation of 500 nt at most were retained as valid hits (3.4 and 4.1 million for Co and MO, respectively). The number of tags matching each mRNA was counted and taken as a measure of the transcript abundance to compute relative abundance of MO. We assumed that most of the genes would be unaffected by the MO and multiplied the MO counts by 0.799 to obtain similar distribution counts between Co and MO. The values for the adjusted lowest, 1^st^ quartile, median, 3^rd^ quartile, and maximum number of tags were 1, 26, 99, 304, and 69,440 for the control and 0.8, 26, 99, 305, and 67,990 for MO, respectively.

Additional technical information about functional analysis of the RNA-seq is given in Protocol S1.

### Antibodies

An affinity-purified polyclonal antibody against xenopus PPARβ was produced by immunizing rabbits with the peptide KLH-VQAPVSDSAAPDSPV (Eurogentec). This antibody detected a 45-kDa protein after immunoblotting of whole-embryo extracts (Fig. S1B). Moreover, a band migrating at the same position was detected when PPAR proteins were pooled-down using a DNA_biotinylated probe containing three copies in tandem of the peroxisome proliferator-activated receptor response element (3xPPRE motif). This band corresponded to PPARβ because PPARα and PPARγ have an expected mass of about 50 kDa and because the efficiency of the pull-down increased when GW501516, a selective PPARβ agonist, was added (Fig. S1B).

We used anti-H3K4me3 from Abcam (ab8580), anti-H3K27me3 from Millipore (07-449), and anti–β-actin from Sigma (AC-40).

### Immunoblotting/DNAP/ChIP

Protein extraction for immunoblotting was done according to the De Robertis online protocol for phospho-proteins (Fuentealba; http://www.hhmi.ucla.edu/derobertis/index.html). DNA affinity purification was done according to [18] starting with the extract from 15 embryos (stg. 12). The DNA probe was produced by annealing the following primers: forward, BioTEG-CGTTCAGGTCAAAGGTCACGTTCAGGTCAAAGGTCACGTTCAGGTCAAAGGTCA and reverse, TGACCTTTGACCTGAACGTGACCTTTGACCTGAACGTGACCTTTGACCTGAACG. GW501516 or DMSO was added together with 10 µl of equilibrated beads, and the extracts were left rotating for 30 min at room temperature before proteins were pulled down and processed for immunoblotting.

ChIP was done according to [19] but using Dynabeads protein G (Invitrogen). For ChIP-reChIP, we proceeded as for ChIP with 150 embryos as starting material. After the TE buffer wash at the end of the first IP, we eluted DNA/protein complexes in 75 µl of TE/10 mM DTT buffer for 30 min at 37°C. The supernatant was diluted 20× with IP buffer. We then proceeded with the second IP, as for a simple ChIP.

Input and immunoprecipitated DNA recovered after de-cross-linking were purified using NucleoSpin Extract II (Macherey-Nagel) and quantified by qPCR.

The percentage of input was calculated as follows:

P_input_ = E_gene_^(**CT**input_gene-**CT**sample_gene)^*100

E stands for gene-specific efficiency and was calculated as explained in the qPCR section below. Enrichment over a negative control was calculated for each primer pair as the ratio of the percentage of input of the IP over the percentage of input of the negative control. For ChIP with H3K4me3 and H3K27me3 antibodies, we used empty beads as negative control. For ChIP with PPARβ antibody, we considered the signal obtained from embryos injected with PPARβ MO as the negative control. Sequences of the primers used are available upon request.

### RT-qPCR

cDNAs were obtained from 500–1000 ng of Trizol-extracted (Invitrogen) RNA using the Quantitect kit (QIAGEN) or SuperScript II (Invitrogen). qPCR runs were processed on an ABI 7900 (Applied Biosystems) or on a Mx3005P (Stratagene) instrument. For each primer pair, a mean PCR efficiency was established *a posteriori* considering all of the amplification curves of that pair, regardless of the condition (Co, MO) with the LinRegPCR program [20]. Relative quantities were calculated by qBase [21] using the single gene efficiency option. Sequences of the primers used are available upon request.

### Screening for H3K27me3 on the promoters of PPARβ promoted genes and control genes

PPAR*β*-promoted genes were chosen among the top 200 most downregulated genes upon MO injection in the list presented in Table S1. The criteria of choice were purely technical: the ability to obtain good primers amplifying a region in the vicinity of the translational start. Similarly, Control genes were taken among genes that did not show a change of expression upon MO injection, according to the RNA-seq. ChIP were done as explained before, but due to the low amount of material in each sample, qPCR were done in duplicate. We considered the tested genes as positive for H3K27me3 when the two following criteria were met: percentage of input >1% and enrichment over mock >5.

## Results and Discussion

### PPARβ is essential for gastrulation movements and antero-posterior axis differentiation in *Xenopus laevis*

In the adult, PPARβ controls many cellular processes that also operate during development [14], [24], which prompted us to investigate its role using a dedicated model. In our eyes *Xenopus laevis* presented many advantages for that study. Besides the ease of embryo manipulation and observation it is noteworthy that PPARβ has been originally identified in that species [25]. An important point is also that PPARβ is highly expressed in the early embryo whereas PPARα is weak and PPARγ absent [25]. This led us to speculate that a major role of PPARβ would not be masked by partially redundant actions of the two other isotypes.

We detected endogenous PPARβ protein in all cell nuclei throughout embryogenesis, with a strong increase during gastrulation to levels that then persisted in subsequent developmental stages (Fig. S1C–L). This pattern of receptor protein expression correlates with mRNA expression [25] and suggests a prominent function for PPARβ during gastrulation.

**Figure 1:**
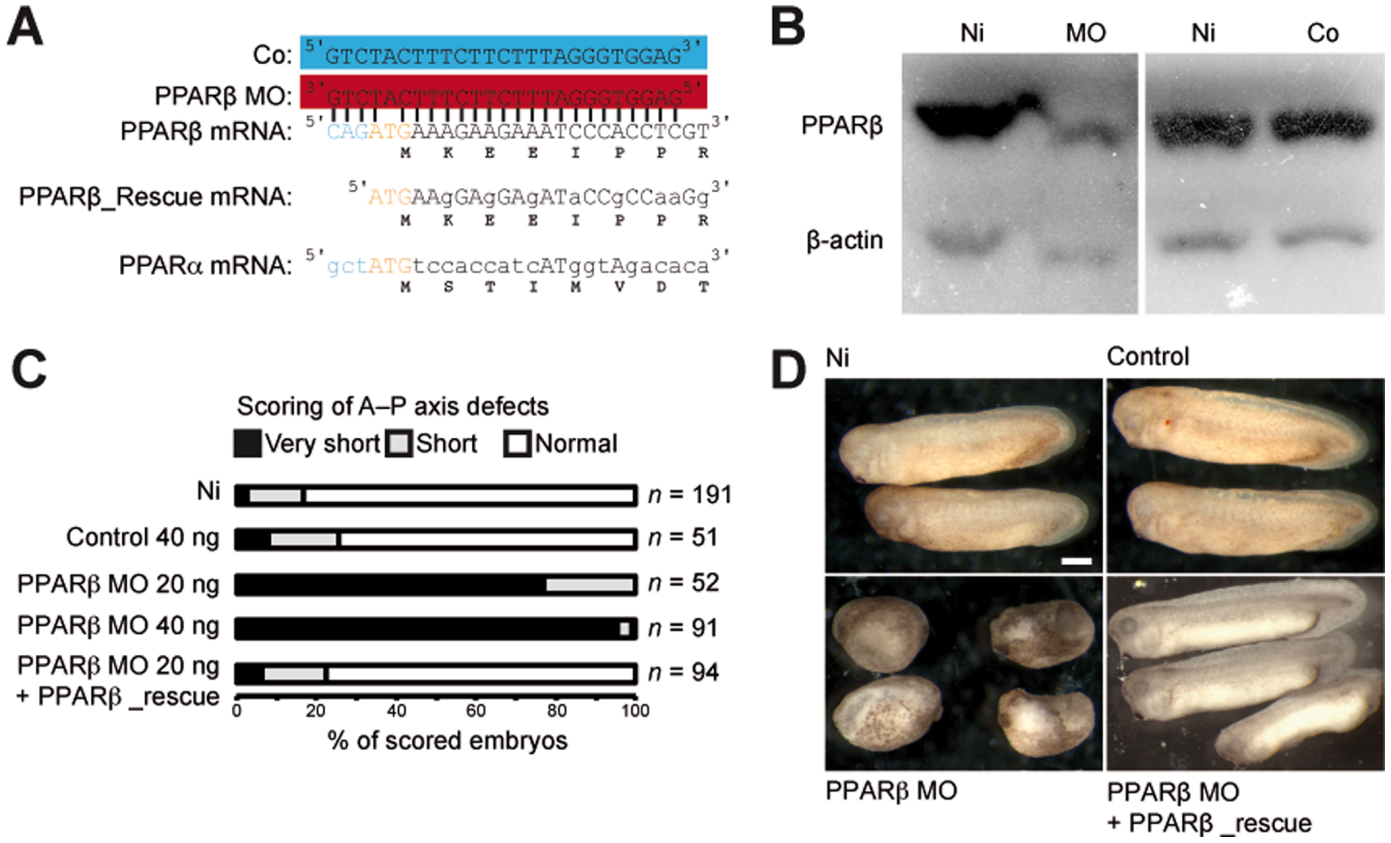
PPARβ is essential for *X. laevis* development. (A) Design of the morpholino (PPARβ MO) to target PPARβ translation and of the control morpholino (Co). Capital letters designate nucleotides that can hybridize with the PPARβ MO. (B) Immunoblot showing endogenous PPARβ levels in non-injected embryos (Ni) and embryos injected with PPARβ MO or Co. β-actin served as a loading control. (C) Scoring of A–P axis defects. Different doses of PPARβ MO, Co, or a combination of PPARβ MO and PPARβ_rescue mRNA were injected. Embryos with a length about a third of that of non-injected sibling embryos were scored as ‘very-short axis’, and those with a length of about two thirds of normal were scored as ‘short axis’. (D) Representative not-injected (Ni), Co-injected (Co), MO-injected (MO), and MO combined with rescue injected (PPARβ MO + PPARβ_rescue) embryos.

This putative role was addressed with a loss-of-function analysis using an antisense morpholino (MO) (Fig. 1A) [26]. PPARβ MO-injected embryos showed a marked decrease in PPARβ protein levels (Fig. 1B), which correlated with a severe reduction in the length of the anterior–posterior axis (Fig. 1C–D). These embryos died shortly after their control siblings reached tail-bud stage. This dramatic phenotype can be entirely attributed to the lack of PPARβ because control morpholino (Co) injection caused no anomaly and PPARβ_Rescue mRNA co-injection together with PPARβ MO restored a normal phenotype (Fig. 1C–D).

**Figure 2:**
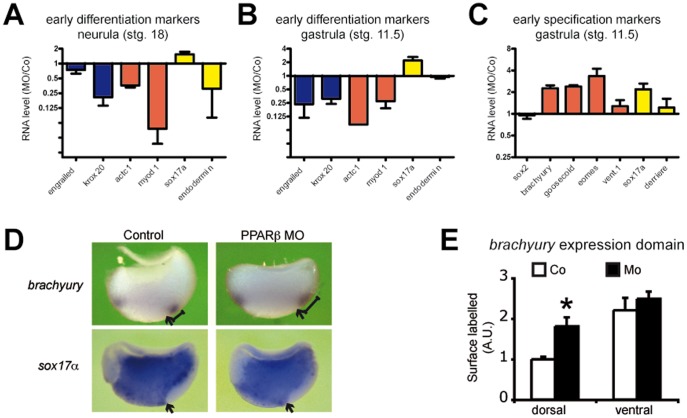
PPARβ promotes differentiation but represses dorsal mesoderm and endoderm specification. (A)–(C) Embryos were injected with PPARβ MO or Co, allowed to develop until stage 18 (A) or stage 11.5 (B) and (D), and collected for extraction of total RNA. qRT-PCR runs for a selection of neural (blue), mesodermal (red), or endodermal (yellow) markers of differentiation (A) and (B) or of germ layer specification (C) were conducted. RNA levels were normalized to EEF1a and RPL8 and are presented as fold variation between MO and Co samples. Error bars represent the S.E.M. of 3 to 5 independent experiments. (D) Embryos were injected with PPARβ MO or Co, fixed at stg. 11.5, hemi-sectioned along the dorso–ventral axis, and processed for RNA *in situ* hybridization. While Mo injection did not affect the *sox17α* expression domain, it resulted in the expansion of *brachyury* expression dorsally (see the scale) but not ventrally. Arrows indicate the dorsal lip. (E) Quantification of the surface covered by the dorsal and ventral expression domains of *brachyury* in MO compared to Co hemi-sections. Error bar is the S.E.M. of 10 measurements. *: two-tailed Student’s t-test vs control, P<0.05.

We verified that PPARβ MO disrupted gastrulation movement by selectively targeting the dorsal marginal zone with a combination of PPARβ MO or Co and fluorescent dye (Fig. S2). At tail-bud stage, PPARβ MO-injected embryos had reduced head and neural tissues and no eyes (Fig. S3). Moreover, a disorganized mass of cells occupied the place of the muscles, and a cell-adhesion defect was obvious (Fig. S3). These results were supported at neurula stage by reduced expression of marker genes consistent with defects in brain (Krox20) and muscle (actc1, myod1) differentiation (Fig. 2A). Of interest, PPARβ already had affected the future neural (engrailed and krox20) and muscle (actc1 and myod1) differentiation before the onset of the gastrulation movements (Fig. 2B).

### Gastrulation is a stage of intense changes in transcript level

**Figure 3:**
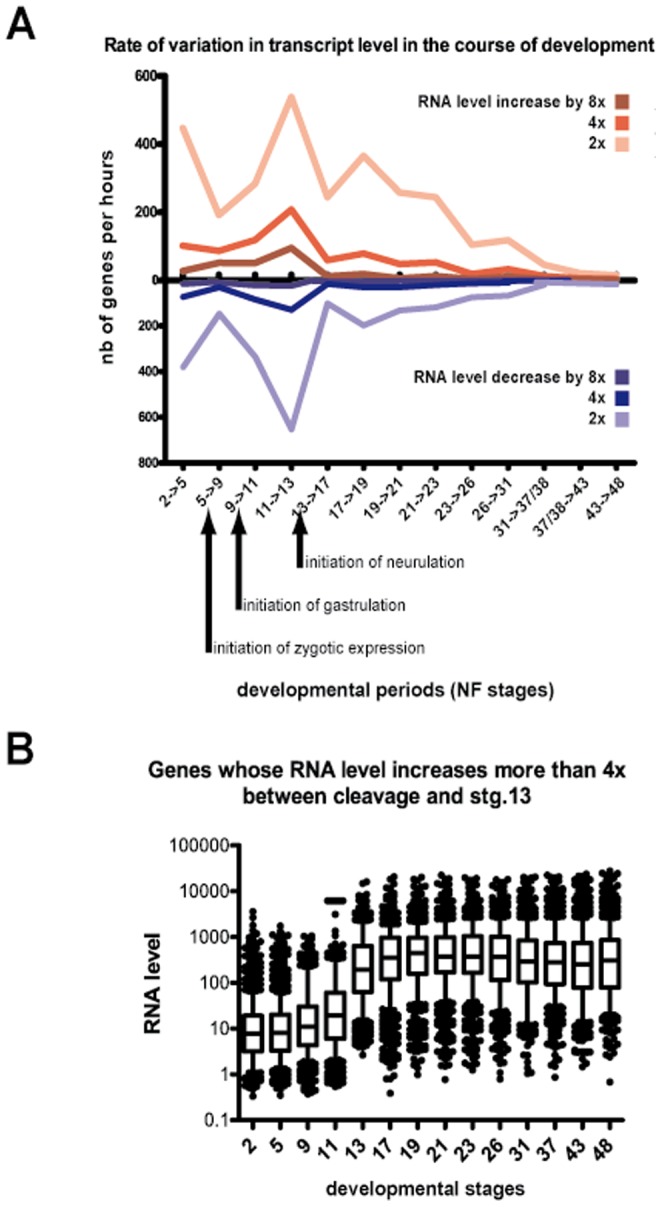
Rate of transcript level variation is maximal at gastrula stage. (A) Data from [23] were used to quantify transcription variations during normal development. The number of genes showing an RNA level increase or decrease by 2×, 4×, or 8× between two consecutive stages was plotted. Data were normalized by the duration, in hours, of each developmental period analysed. (B) The group of genes with RNA levels that increased 4× or more between stage 11 and stage 13 was considered, and the RNA levels of these genes were plotted at different developmental stages. The rectangles delineate the 25^th^ and 75^th^ percentiles, the horizontal bar is the median, and the whiskers indicate the 10^th^ and 90^th^ percentiles.

We evaluated the relevance of this early gastrula stage control of differentiation marker expression by using data available from a recent transcriptomic report [23]. We determined changes in the level of many transcripts during normal *X. laevis* development. Based on analysis of a period ranging from cleavage to the feeding tadpole stages, the transition from mid- to late gastrula corresponded to the stage of highest variations in individual transcript levels, whether an increase or decrease (Fig. 3A). The genes that were strongly activated during gastrulation maintained their higher level of expression throughout subsequent stages (Fig. 3B), indicating that expression changes at gastrula were not limited to genes solely required for gastrulation itself but also included several potential differentiation regulators.

### Transcriptomic analysis of PPARβ knock-down

**Figure 4:**
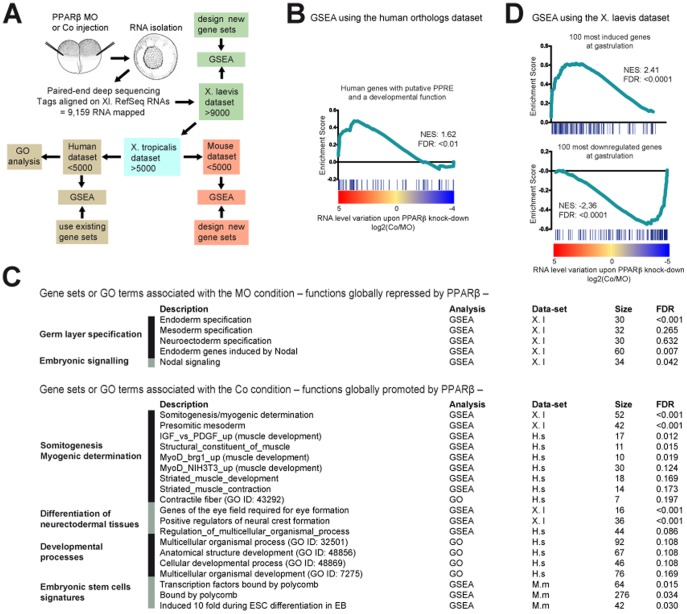
PPARβ promotes the initiation of differentiation at gastrulation. (A) Rationale of the transcriptomic analysis of PPARβ loss-of-function. (B) The gene set consisting of predicted direct PPAR target genes in humans [33] was analysed by GSEA. (C) The Gene Ontology terms or the gene sets that were significantly (FDR<0.2) affected by PPARβ loss-of-function are presented. The gene sets corresponding to germ layer specification are also presented. (D) The gene sets consisting of the 100 most-induced genes and of the 100 most-decreased genes at gastrula (see also Fig. S5) were analysed by GSEA. FDR, false discovery rate; GSEA, Gene Set Enrichment Analysis; NES, Normalized enrichment score.

Because of the importance of this stage in the reorganization of the transcription profile, we analysed the impact of PPARβ on the transcriptome by RNA-seq (Fig. 4A, Table S1 and Fig. S4, and Methods). A clear PPAR signature was revealed; most of the *X. laevis* orthologs of human genes with a predicted peroxisome proliferator-activated receptor response element were stimulated by PPARβ at mid-gastrula (Fig. 4B), suggesting conservation of PPAR function between frogs and mammals. The RNA-seq data also confirmed the histology and marker gene expression presented above, i.e., PPARβ promotes developmental functions related to muscle and neural differentiation (Fig. 4C). Of importance, neuroectoderm and mesoderm specification was not reduced by the lack of PPARβ, and the latter was even increased dorsally (Fig. 2C–E and Fig. 4C). Thus, we excluded the possibility that down-regulation of differentiation genes was the indirect consequence of a lack of germ layer induction. Collectively, our analyses identified PPARβ as a major promoter of differentiation *in vivo*. In support of this finding, PPARβ controlled the expression of the majority of the genes whose RNA level varies the most between the cleavage and gastrula stages, be it a rise or a decline of expression (Fig. 4D). In fact, this finding indicates a novel, unsuspected role of PPARβ in governing a massive wave of transcriptional modifications initiated at early gastrulation and affecting the later differentiation of organs such as muscle or brain. Embryogenesis in PPARβ-null mice has not been studied in detail, possibly because defects in placenta formation may complicate the analysis [15]; however, no gross gastrulation or differentiation defects have been reported to date. This gap suggests that either PPARβ functions in early mouse embryogenesis are masked by redundancy with other factors or that PPARβ has specialized to control cell differentiation in the placenta during evolution.

### Chromatin signature of PPARβ target genes at gastrulation

Epigenetic mechanisms, such as those described in ESCs, could facilitate such a coordinated broad change in the transcription profile [5], [9]. Of great interest, for many genes that PPARβ activated, the mouse orthologs are strongly activated during ESC differentiation [3] (Fig. 4C). Moreover, PPARβ also stimulated genes, which mouse orthologs are bound by the polycomb repressive complex that is known to deposit H3K27me3 marks in ESCs [3] (Fig. 4C). This association led us to hypothesize cooperation between PPARβ and epigenetic marks. We classified *X. laevis* genes into two groups based on the epigenetic signatures of mouse and/or zebrafish orthologs in pluripotent cells (Discussion S1) [8], [9]: first, the ‘K27’ group of genes, marked by H3K27me3 regardless of co-occupancy with H3K4me3, and second, the ‘K4 only’ group, marked by H3K4me3 but not by H3K27me3. Strikingly, PPARβ promoted transcription of a significant fraction of ‘K27’ genes but of very few ‘K4 only’ genes (Fig. S5). This finding suggested an influence of chromatin marks on PPARβ responsiveness, which was even more pronounced when we considered only the 100 genes whose RNA level increased the most during *X. laevis* gastrulation (same set of genes as in Fig. 4D). PPARβ enhanced expression of 81 of these genes, 44 of which were ‘K27’ and 25 of which were ‘K4 only’ (Fig. S5B). Reciprocally, PPARβ repressed 71 out of the 100 most-decreased transcripts at gastrula, and most of these repressed genes (50 genes) were of the ‘K4 only’ class (Fig. S5B). Of note, *X. laevis* putative ‘K27’ genes were strongly activated very early before the end of gastrulation while ‘K4 only’ genes were mostly maternal with high, relatively steady RNA levels over time (Fig. S6). Because of the possible presence of maternally inherited transcripts for the ‘K4 only’ genes, it was not possible to separate these sets of genes in inactive and active genes at gastrulation. All together, the data indicated that, *Xenopus* orthologs of ESCs genes marked by H3K27me3 are synchronously induced at gastrula and globally promoted by PPARβ. On the contrary, *Xenopus* orthologs of genes marked by H3K4me3 are globally repressed by PPARβ with their RNA level being stable or in slight decrease over gastrulation. In ESCs, genes marked by H3K27me3 during pluripotent stage are globally induced during ESC differentiation, while H3K4me3 genes become repressed [1]. Therefore our data show that *X*. *laevis* gastrulation is transcriptionally closely related to ESC differentiation, which was not anticipated. Is there a mechanism of synchronous activation that is conserved in *Xenopus* and mouse? Could it be that an epigenetic signature of pluripotency is established in the course of *X. laevis* early development and involves orthologs of genes marked in ESCs?

Based on present knowledge, this would be surprising because (i) epigenetic signatures of pluripotency are poorly conserved across species in general (even between mammals) and (ii) *X. laevis* is thought to be deprived of them (Discussion S1) [27].

This prompted us to explore the possibility that H3K27me3 and H3K4me3 are deposited during the pluripotent stages of early *X. leavis* development and that PPARβ cooperates with these epigenetic marks later during gastrulation.

### Identification of bivalent genes as a chromatin signature of pluripotency in *Xenopus laevis* blastula

We reasoned that if epigenetic marks would be conserved between zebrafish and mouse they might also be in *X. laevis*. Therefore, to challenge the notion of a collaboration of PPARβ with epigenetic marks we started by defining a set of *X*. *laevis* genes for which the ‘K27’ or ‘K4 only’ status of their orthologs is conserved in both fish blastulae and mouse ESCs.

**Figure 5:**
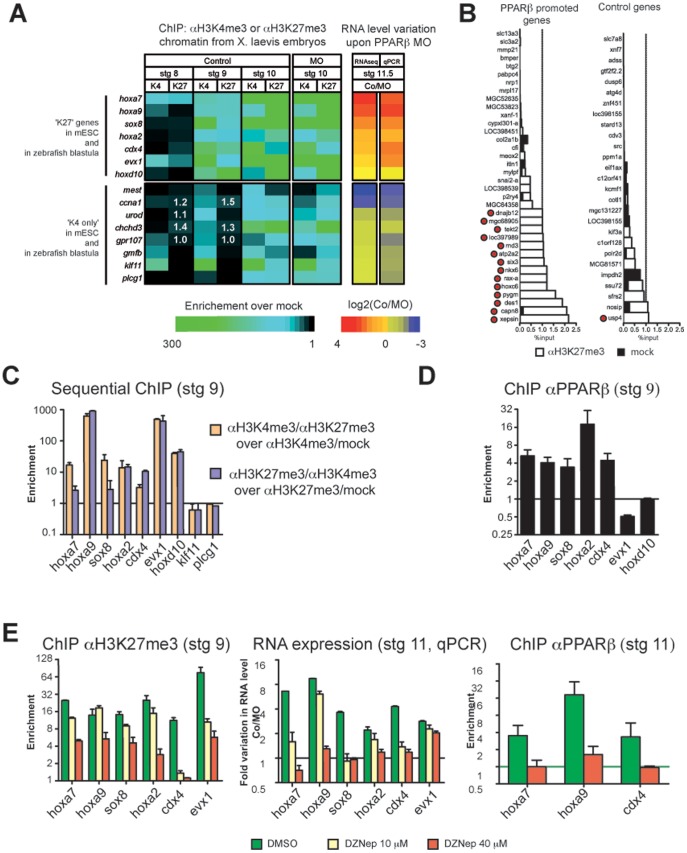
PPARβ interprets a chromatin signature that is deposited at the end of the pluripotent stage. (A) Seven ‘K27’ genes and eight ‘K4 only’ genes were analysed by ChIP as indicated. Results are presented in a heat map (see also Supplementary Fig. 7). Variation in RNA expression upon PPARβ MO injection was obtained from the RNA-seq data or from qPCR validations. (B) ChIP with H3K27me3 antibody was conducted at stage 9 on 37 ‘PPARβ promoted genes’ and on 27 Control genes. PPARβ promoted genes were chosen among the top 200 most downregulated genes at stage 11, upon MO injection in the list presented in Table S1, while Control genes did not show a change of expression upon MO injection. Results are presented as percentage of input. The threshold of 1% is indicated. Genes scored as positive for H3K27me3 are indicated by a red dot (see methods for further details on the definition of gene sets and on the criteria of scoring). (C) Sequential ChIPs were conducted. Note that no enrichment was observed for klf11 and for plcg1, which represent negative controls (see panel b). Error is the S.E.M of 2 independent experiments. (D) ChIP using PPARβ antibody was conducted at stage 11.5. Error is the S.E.M of 3 to 4 independent experiments. (E) ChIP with H3K27me3 antibody or PPARβ antibody and *q*RT-PCR were conducted on embryos treated with DZNep or DMSO and injected with PPARβ MO or Co. Error is the S.E.M of technical replicates of a single experiment that we have replicated with similar results.

15 *X. laevis* genes of this set were tested for H3K27me3 and H3K4me3 marks at different developmental stages from blastula (pluripotent stage) to mid-gastrula. From blastula stage onwards, both H3K27me3 and H3K4me3 marks showed a progressive accumulation (Fig. 5A and Fig. S7), which is consistent with previous reports [27], [28]. However, in contrast to what has been proposed [27], [28], we saw no delay in H3K27me3 accumulation as compared to H3K4me3. Of greatest importance, at stage 9 (late blastula), our data fit very well with the predictions derived from ESC and zebrafish blastulae (Fig. 5A). Indeed, seven of the seven genes predicted to be ‘K27’ displayed a clear H3K27me3 signal at this stage. These genes were also all enriched in H3K4me3, consistent with the bivalent status of their orthologs in fish and ESCs. Similarly, seven out of the eight predicted ‘K4 only’ genes were indeed ‘K4 only’.

These results suggested the occurrence of bivalent genes in frogs, in contrast to previous findings [27]. We therefore tested the seven validated ‘K27/K4’ genes by sequential ChIP at the end of the blastula stage (stg. 9) and found that they were all bivalent (Fig. 5C). Thus, we show for the first time that *X. laevis* late blastula cells resemble pluripotent ESCs and fish blastula cells with respect to H3K4me3 and H3K27me3 marks. We conclude that this epigenetic signature of pluripotency is present in frogs, and might thus be a common feature of vertebrates.

### H3K27me3 marks serve as cues for activation by PPARβ at gastrula

What is most important from this work is the finding that validated ‘K27’ genes encoded transcripts that showed an abrupt increase at gastrula stage (Fig. S6C) were promoted by PPARβ at this stage (Fig. 5A). Moreover, five of these genes are most likely direct PPARβ target genes because PPARβ occupied their chromatin at this stage (Fig. 5D). Thus, PPARβ and polycomb complex activity converge to promote transcription of early developmental regulators at gastrulation. This conclusion raised the question of any functional interdependence between these two pathways. Analysis of H3K4me3 and H3K27me3 marks in PPARβ-depleted embryos revealed no major changes compared to the control at stage 10.5 (Fig. 5A). PPARβ therefore does not affect H3K27me3 or H3K4me3 deposition, excluding the possibility that the decreased RNA levels observed for ‘K27’ genes upon PPARβ MO injection resulted from differences in the level of activating or repressing epigenetic marks. Rather, PPARβ appears to read the epigenetic status of target genes and preferentially activate the ‘K27’ genes.

However, this conclusion is based on only 6 *X*. *laevis* genes that are part of a subset of ‘ultra-conserved’ genes with respect to pluripotency signature. We thus wanted to test additional genes with no a priori on their epigenetic state. We reasoned that if PPARβ preferentially activated K27 genes, then there should be more K27 genes among genes that are promoted by PPARβ at gastrula than among genes that are not affected by PPARβ MO. To test this idea, we screened for the K27 mark at late blastula (see Material and Methods) on a set of genes that are later strongly activated by PPARβ and on a set of control genes. 14 out of the 35 PPARβ target genes were scored positive for K27, whereas only one out of the 27 control genes was K27 positive (Fig.5B; Fisher exact test, P<0.005). The low level of K27 in control genes (less than 4%) is consistent with the low level of H3K27me3 marks observed by mass spectrometry at this stage [29]. Finally, when results presented in Fig. 5A and B are taken together, we have identified 22 K27 genes on a total of 78 genes investigated, and 20 of them are promoted by PPARβ. This is a clear support of our hypothesis that PPARβ collaborates with K27 mark to promote gene expression.

To get more insights into this collaboration, we combined MO injection with treatment of developing embryos with DZNep, a drug that inhibits the activity of polycomb repressive complex 2 in mammals [30]. This treatment was efficient at limiting H3K27me3 deposition in frog embryos (Fig. 5E). At the highest dose of DZNep, PPARβ no longer promoted transcription of its direct ‘K27’ targets genes (hoxa7, hoxa9, sox8, hoxa3, and cdx4) (Fig. 5E). However, for evx1, which is not a PPARβ direct target gene (Fig. 5D), the indirect PPARβ effect remained in spite of a 13× reduction in H3K27me3 level at this locus (Fig. 5E). Finally, for hoxa7, hoxa2 and cdx4, PPARβ binding was abrogated in the presence of the highest dose of DZNep (Fig. 5E), which suggests that at least for these genes, the H3K27me3 mark contributes to the recruitment or stabilization of PPARβ on their promoter region at gastrulation.

Collectively, these data provide a proof of concept that in vivo, a transcription factor can recognize chromatin status established at pluripotent stage and modulate its activity accordingly: epigenetic marks deposited at the end of the pluripotent stage (late blastula) can influence later gene expression at gastrula under the control of PPARβ. In particular, our data challenge the interpretation that H3K27me3 can counteract only an activating effect of the H3K4me3 mark on bivalent genes [3], [5]; instead, they show that H3K27me3 can serve as a cue to stimulate gene transcription. At first sight, this finding seems to contradict the well-documented role of H3K27me3 in gene repression; however, it parallels previous observations that in the course of mouse ESC differentiation, some genes are activated even if their promoter is occupied by polycomb proteins and clearly marked by H3K27me3 [10]. It is also consistent with the phenotype of mice and of *Xenopus* embryos lacking PRC2 function that show delayed or impaired induction of early differentiation genes [28], [31], [32]. The present works shows that *X*. *laevis* can be a model of choice to study the relation between epigenetic marks and the switch between pluripotency and differentiation in vivo. *X. laevis* blastulae and gastrulae cells are somehow comparable to pluripotent and differentiating ESCs, respectively. Furthermore, the identification of PPARβ as a crucial factor that interprets the chromatin signature of pluripotency in this system could serve as a hook for further mechanistic investigation.

## Supporting Information

Figure S1 **A dedicated peptide-derived antibody detects endogenous PPARβ protein throughout** ***Xenopus laevis*** **early development.** (A) Multiple alignments of the protein sequences of xPPARβ, xPPARα, and hPPARβ/δ. The region in blue, which corresponds to the peptide used to generate the xPPARβ antibody, is not conserved. (B) DNA affinity purification of gastrula extracts using a 3× peroxisome proliferator-activated receptor response element biotinylated probe in the presence of increasing concentrations of the PPARβ agonist GW501516. The lane labelled “embryo extract” corresponds to the input. (C) Immunoblot showing endogenous levels of PPARβ protein in total embryo extracts. β-actin is shown as a loading control. Numbers refer to developmental stages. The arrows mark the beginning of the indicated phases. (D)–(L) Immunolocalization of endogenous PPARβ protein. Sections of gastrula (stg. 11; D–I) and early tailbud (stg. 31; J–L) processed to immunolocalize endogenous PPARβ and observed by fluorescence microscopy are presented. (D) PPARβ signal. (F) DAPI signal obtained from the same section. (H) Overlay of the PPARβ and DAPI signals. (E), (G), (I), Close-ups of (D) f, and h, respectively, showing nuclear localization. Similarly, (J), (K), and (L) were obtained from the same section and represent the PPARβ signal, the DAPI signal, and the overlay of both signals, respectively. Scale bar is 500 µm in (H) and (L) and 100 µm in (I).
(TIF)

Figure S2 **PPARβ promotes gastrulation movements.** (A) Rationale of the experiment. Eight-cell–stage embryos were injected in one dorsal animal blastomere with a solution of fluorescent Texas Red® dextran mixed with Co or PPARβ MO. Embryos were allowed to develop until the neurula stage, when they were observed using a microscope set to detect Texas Red® fluorescence (B) and (E). Embryos where then sectioned either along a sagittal plane (C) and (F) or a transverse plane (D) and (G). (B) and (E) represent the overlay of the bright-field and Texas Red® channels. (C) (D) (F), and (G) images are composed with the overlay of the DAPI (light blue), Texas Red® (red), and bright-field (grey) channels. Scale bar is 500 µm. DMZ: dorsal marginal zone. When gastrulation movements are well advanced, the Co-containing cells were distributed in a narrow strip all along the midline, as expected (B)–(D). On the contrary, PPARβ MO-containing cells were packed together with no apparent migration phenotype (E)–(G). We conclude that PPARβ promotes gastrulation movements.
(TIF)

Figure S3 **Histological analyses of PPARβ loss-of-function embryos.** (A) and (B) are sagittal sections of specimens presented in Fig. 1d, stained with haematoxylin–eosin. (C) and (D) PPARβ MO alone (C) or combined with PPARβ_rescue mRNA (D) was injected into one blastomere of the two-cell–stage embryo (unilateral injection). Note that the embryos unilaterally injected with PPARβ MO were curved because of an asymmetric elongation of the A–P axis. Longitudinal sections were stained with haematoxylin–eosin.
(TIF)

Figure S4 **Validation of the transcriptomic analysis of PPARβ knockdown at mid-gastrula.** (A) and (B) For 30 transcripts, the relative expression in PPARβ MO vs Co obtained from the same stage of development (11.5) was compared between RNA-seq and qPCR. Data are presented in a table (A) and in a correlation plot (B).
(TIF)

Figure S5 **PPARβ activity differs depending on putative epigenetic marks.** (A) The H3K4me3 and H3K27me3 state of genes in mouse ESC (mESC) (2 first columns at the left [5]) or in zebrafish (the 2 columns in the middle [8]) was used to infer a ‘K4 only’ or a ‘K27’ state of *X. laevis* orthologs. For these genes, the variations in RNA level induced by PPARβ depletion (RNA-seq data) are presented as a heat map. Red genes are promoted by PPARβ while blue genes are repressed at stage 11.5. The two columns on the right represent the genes for which the epigenetic state is conserved between mESCs and zebrafish blastulae. (B) Venn diagrams showing the overlap between PPARβ activity, chromatin signature (refer to the main text for the definition of the classes), and expression profile of *X. laevis* genes at gastrulation.
(TIF)

Figure S6 **‘K27’ and ‘K4 only’ genes have distinct kinetics of expression.** The expression profile of ‘K27’ and ‘K4 only’ genes (see the main text for the description of the classes) is presented at different stages of *X. laevis* development using data from [23]. (A) Genes inferred from mouse ESC (mESC) data; (B) genes inferred from zebrafish data; (C) conserved ‘K4 only’ and ‘K27’ genes in zebrafish and mESC. Bold blue lines in c correspond to validated ‘K4 only’ genes while red lines are for validated ‘K27’ genes. In (A) and (B) the rectangles delineate the 25^th^ and 75^th^ percentiles, the horizontal bar is the median, and the whiskers indicate the 10^th^ and 90^th^ percentiles.
(TIF)

Figure S7 **Individual data from ChIP experiments presented in** **Figure 5B** **.** ChIP data obtained with the H3K4me3 antibody are presented in red and those obtained with the H3K27me3 antibody are in green. Each point represents an independent experiment.
(TIF)

Table S1 (XLS)

Table S2 (XLS)

Protocol S1 **Functional analysis of RNA-seq data and Most-induced and most-decreased transcripts at gastrulation definition.**
(DOCX)

Discussion S1 **Choice of epigenetic signatures of pluripotency.**
(DOCX)
