# Supplementary figures and images for: PPARβ Interprets a Chromatin Signature of Pluripotency to Promote Embryonic Differentiation at Gastrulation
Source: PLoS One. 2013 Dec 18;8(12):e83300. doi: 10.1371/journal.pone.0083300 (PMC3867458; doi:10.1371/journal.pone.0083300)

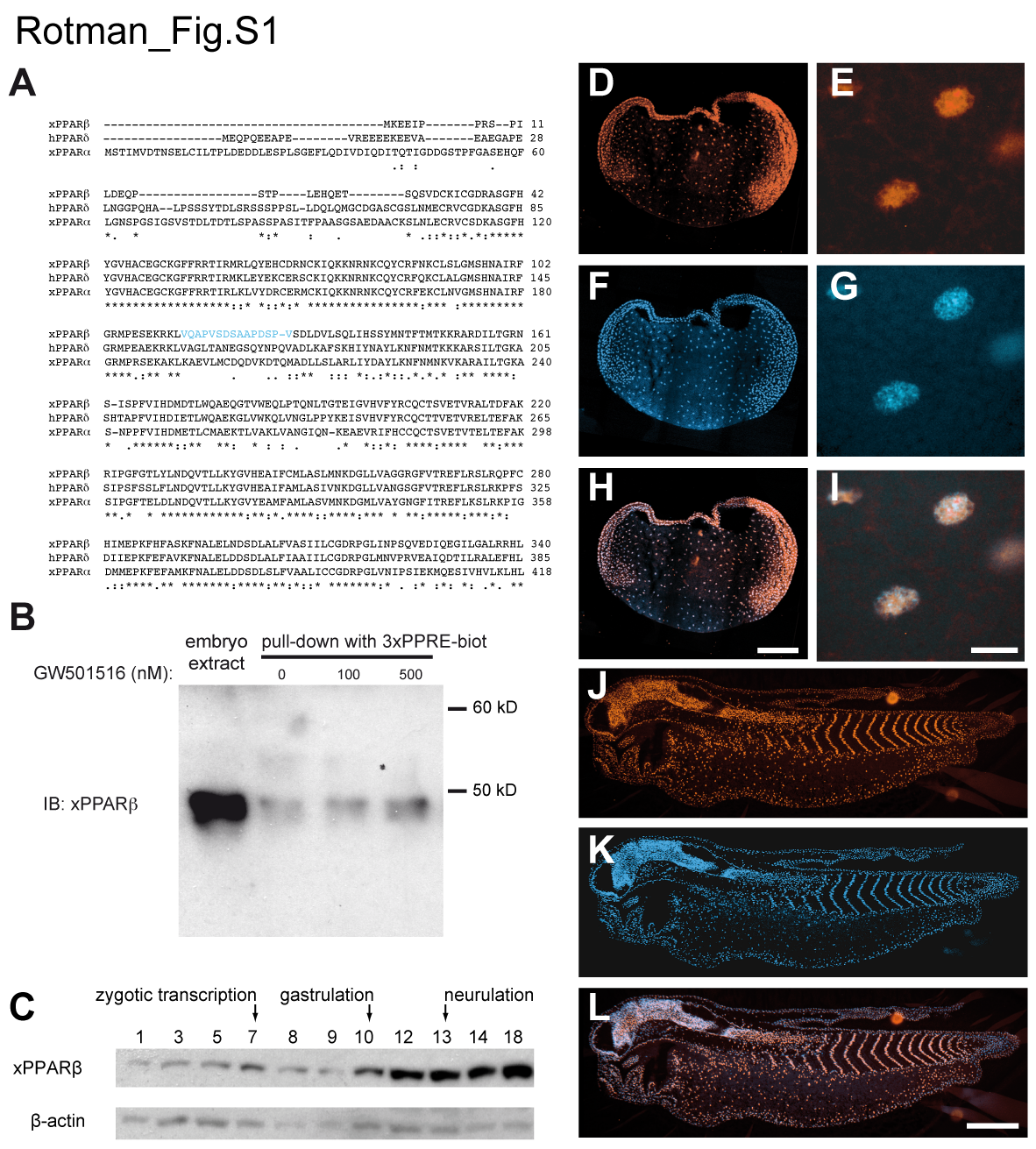

Supplement: Figure S1 — A dedicated peptide-derived antibody detects endogenous PPARβ protein throughout Xenopus laevis early development. (A) Multiple alignments of the protein sequences of xPPARβ, xPPARα, and hPPARβ/δ. The region in blue, which corresponds to the peptide used to generate the xPPARβ antibody, is not conserved. (B) DNA affinity purification of gastrula extracts using a 3× peroxisome proliferator-activated receptor response element biotinylated probe in the presence of increasing concentrations of the PPARβ agonist GW501516. The lane labelled “embryo extract” corresponds to the input. (C) Immunoblot showing endogenous levels of PPARβ protein in total embryo extracts. β-actin is shown as a loading control. Numbers refer to developmental stages. The arrows mark the beginning of the indicated phases. (D)–(L) Immunolocalization of endogenous PPARβ protein. Sections of gastrula (stg. 11; D–I) and early tailbud (stg. 31; J–L) processed to immunolocalize endogenous PPARβ and observed by fluorescence microscopy are presented. (D) PPARβ signal. (F) DAPI signal obtained from the same section. (H) Overlay of the PPARβ and DAPI signals. (E), (G), (I), Close-ups of (D) f, and h, respectively, showing nuclear localization. Similarly, (J), (K), and (L) were obtained from the same section and represent the PPARβ signal, the DAPI signal, and the overlay of both signals, respectively. Scale bar is 500 µm in (H) and (L) and 100 µm in (I). (TIF) [file pone.0083300.s001.tif]

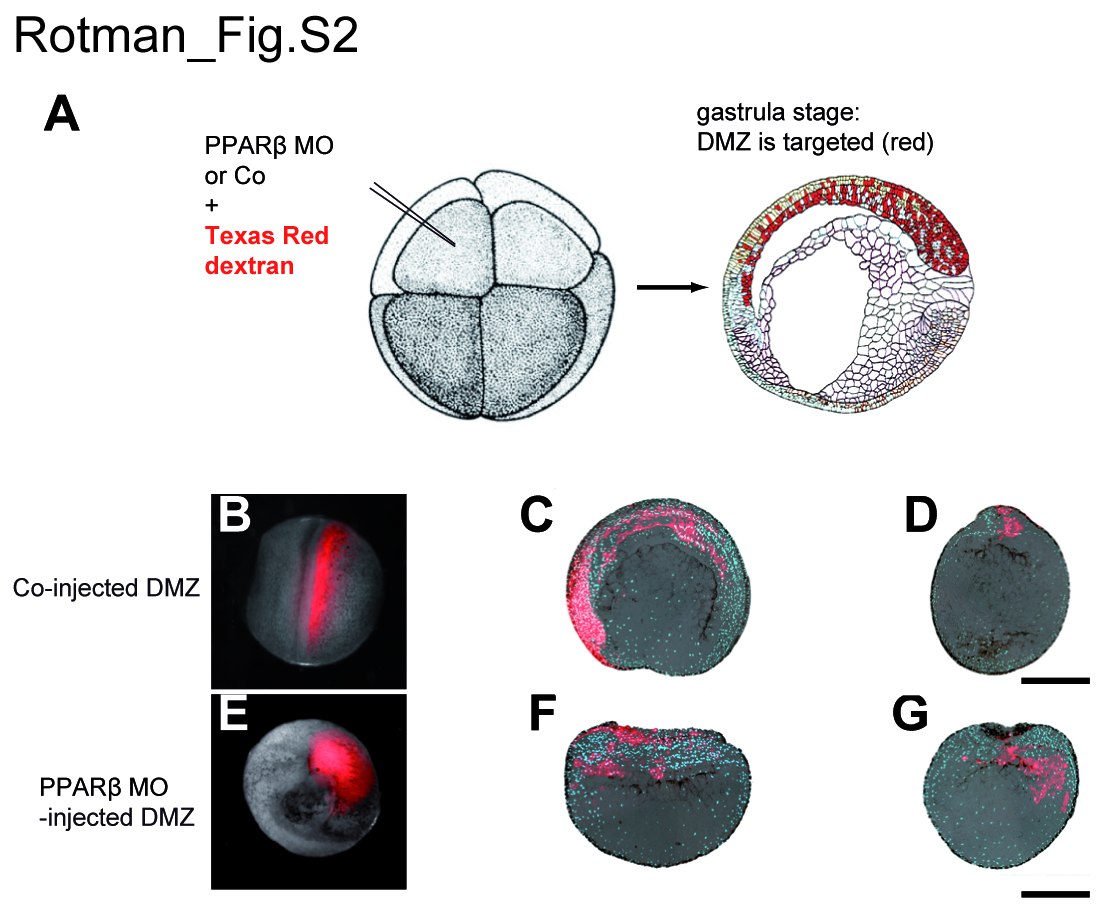

Supplement: Figure S2 — PPARβ promotes gastrulation movements. (A) Rationale of the experiment. Eight-cell–stage embryos were injected in one dorsal animal blastomere with a solution of fluorescent Texas Red® dextran mixed with Co or PPARβ MO. Embryos were allowed to develop until the neurula stage, when they were observed using a microscope set to detect Texas Red® fluorescence (B) and (E). Embryos where then sectioned either along a sagittal plane (C) and (F) or a transverse plane (D) and (G). (B) and (E) represent the overlay of the bright-field and Texas Red® channels. (C) (D) (F), and (G) images are composed with the overlay of the DAPI (light blue), Texas Red® (red), and bright-field (grey) channels. Scale bar is 500 µm. DMZ: dorsal marginal zone. When gastrulation movements are well advanced, the Co-containing cells were distributed in a narrow strip all along the midline, as expected (B)–(D). On the contrary, PPARβ MO-containing cells were packed together with no apparent migration phenotype (E)–(G). We conclude that PPARβ promotes gastrulation movements. (TIF) [file pone.0083300.s002.tif]

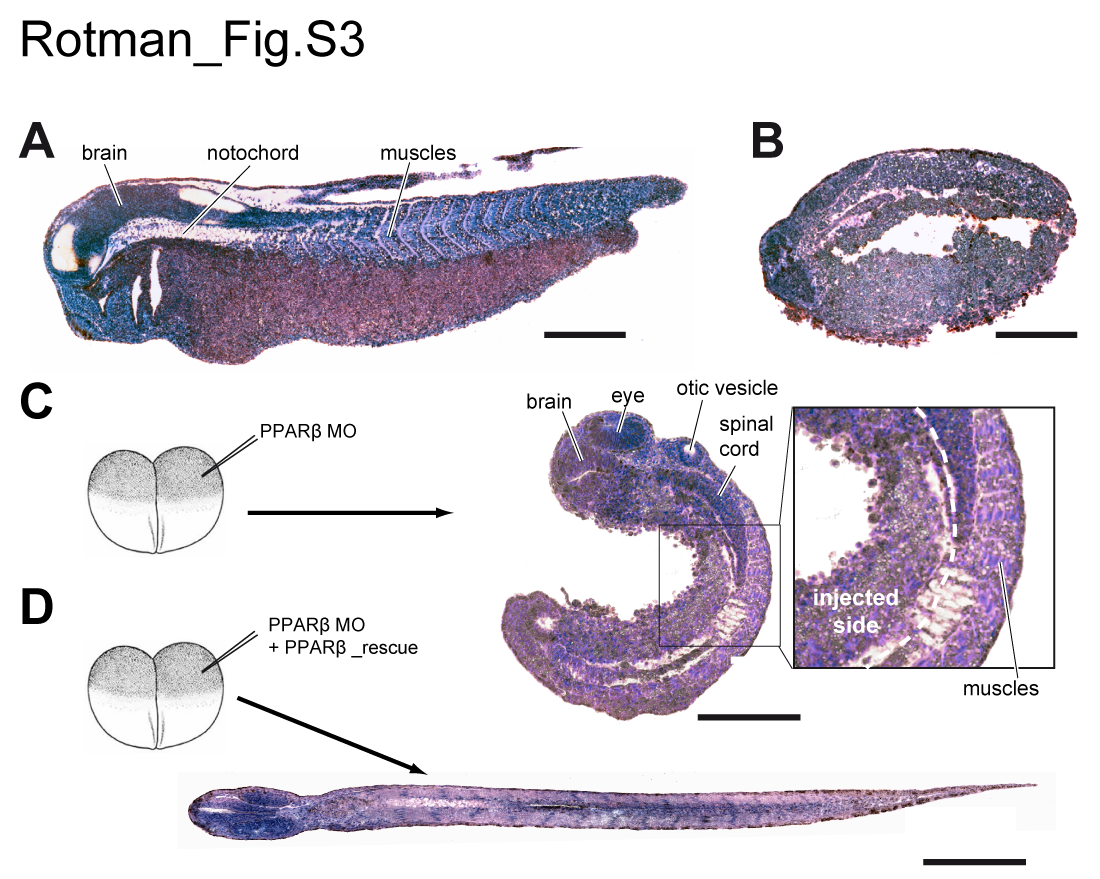

Supplement: Figure S3 — Histological analyses of PPARβ loss-of-function embryos. (A) and (B) are sagittal sections of specimens presented in Fig. 1d, stained with haematoxylin–eosin. (C) and (D) PPARβ MO alone (C) or combined with PPARβ_rescue mRNA (D) was injected into one blastomere of the two-cell–stage embryo (unilateral injection). Note that the embryos unilaterally injected with PPARβ MO were curved because of an asymmetric elongation of the A–P axis. Longitudinal sections were stained with haematoxylin–eosin. (TIF) [file pone.0083300.s003.tif]

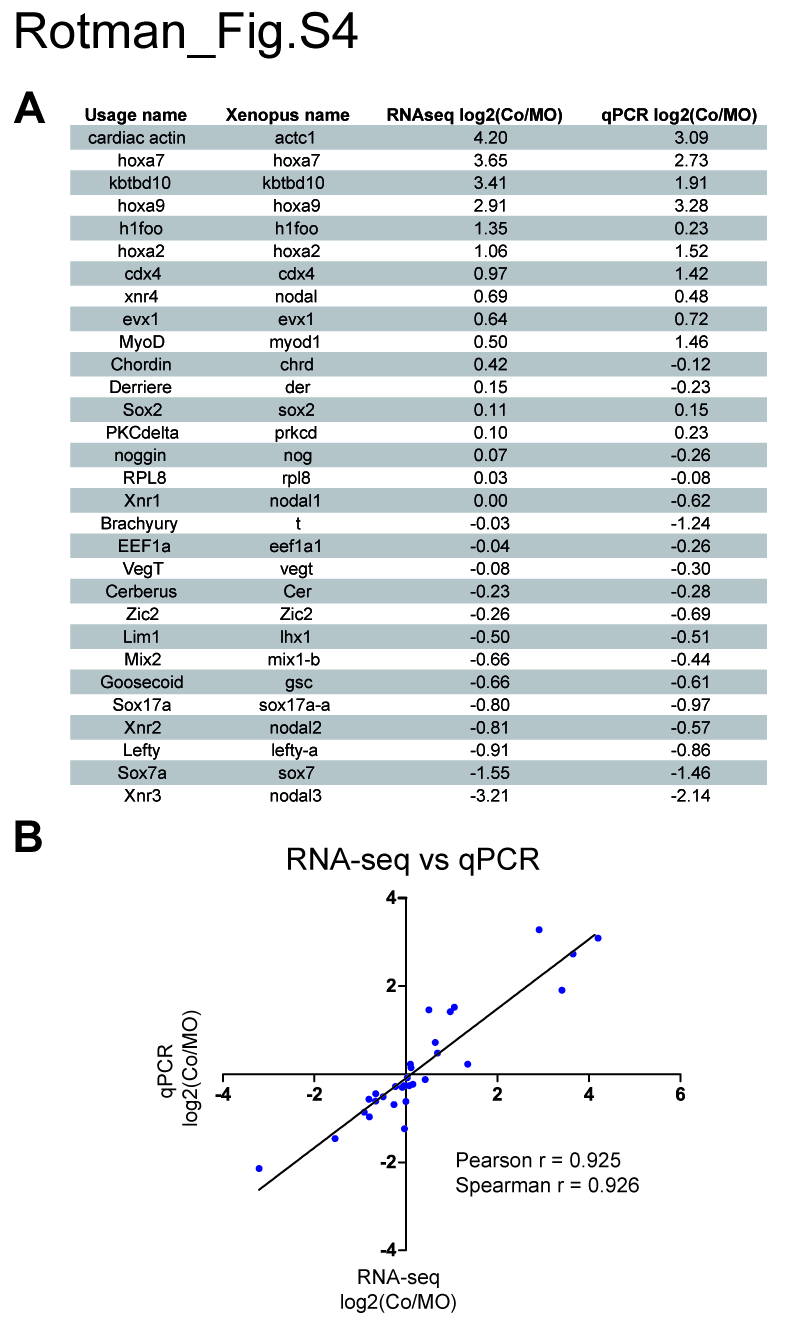

Supplement: Figure S4 — Validation of the transcriptomic analysis of PPARβ knockdown at mid-gastrula. (A) and (B) For 30 transcripts, the relative expression in PPARβ MO vs Co obtained from the same stage of development (11.5) was compared between RNA-seq and qPCR. Data are presented in a table (A) and in a correlation plot (B). (TIF) [file pone.0083300.s004.tif]

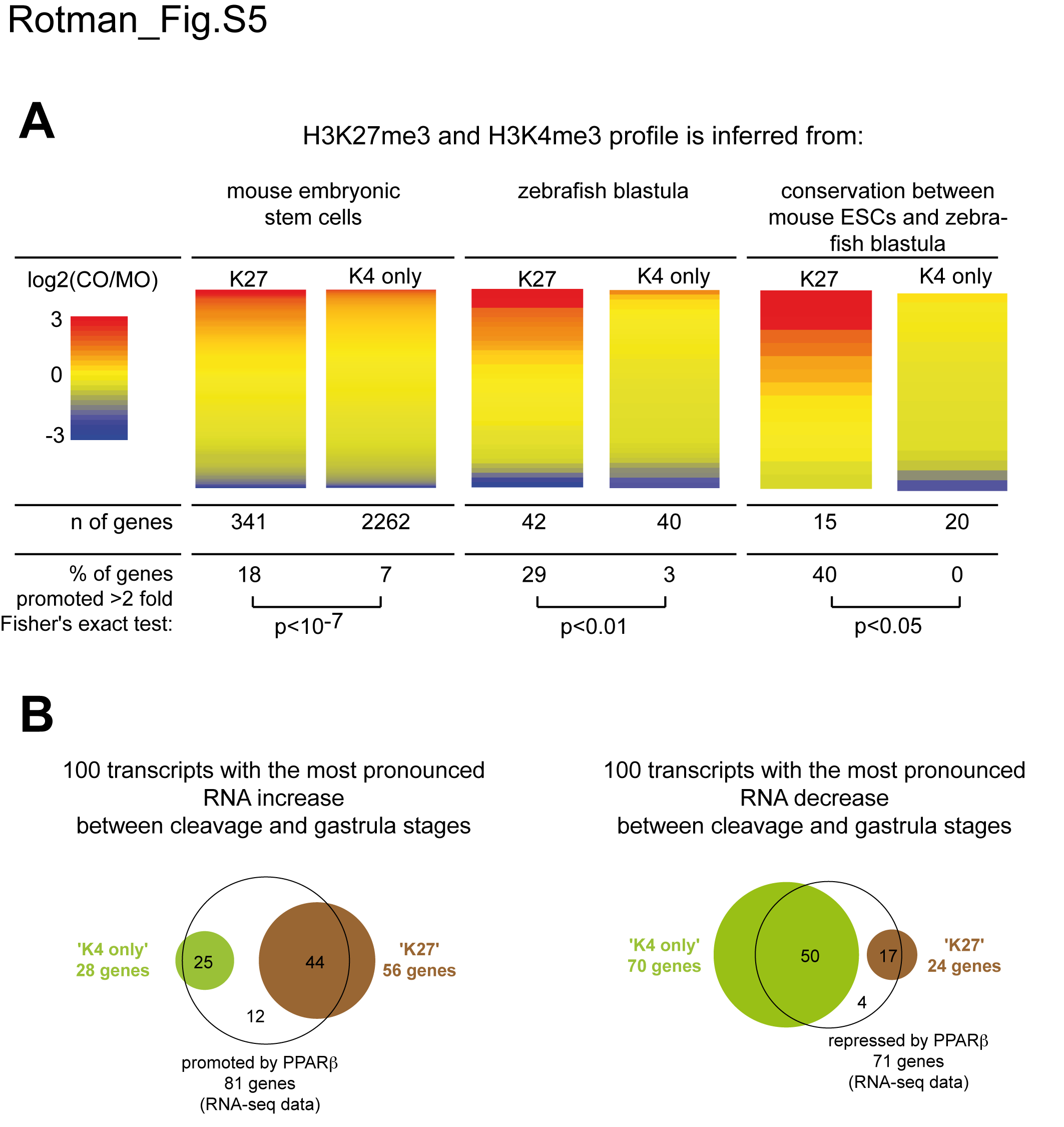

Supplement: Figure S5 — PPARβ activity differs depending on putative epigenetic marks. (A) The H3K4me3 and H3K27me3 state of genes in mouse ESC (mESC) (2 first columns at the left [5]) or in zebrafish (the 2 columns in the middle [8]) was used to infer a ‘K4 only’ or a ‘K27’ state of X. laevis orthologs. For these genes, the variations in RNA level induced by PPARβ depletion (RNA-seq data) are presented as a heat map. Red genes are promoted by PPARβ while blue genes are repressed at stage 11.5. The two columns on the right represent the genes for which the epigenetic state is conserved between mESCs and zebrafish blastulae. (B) Venn diagrams showing the overlap between PPARβ activity, chromatin signature (refer to the main text for the definition of the classes), and expression profile of X. laevis genes at gastrulation. (TIF) [file pone.0083300.s005.tif]

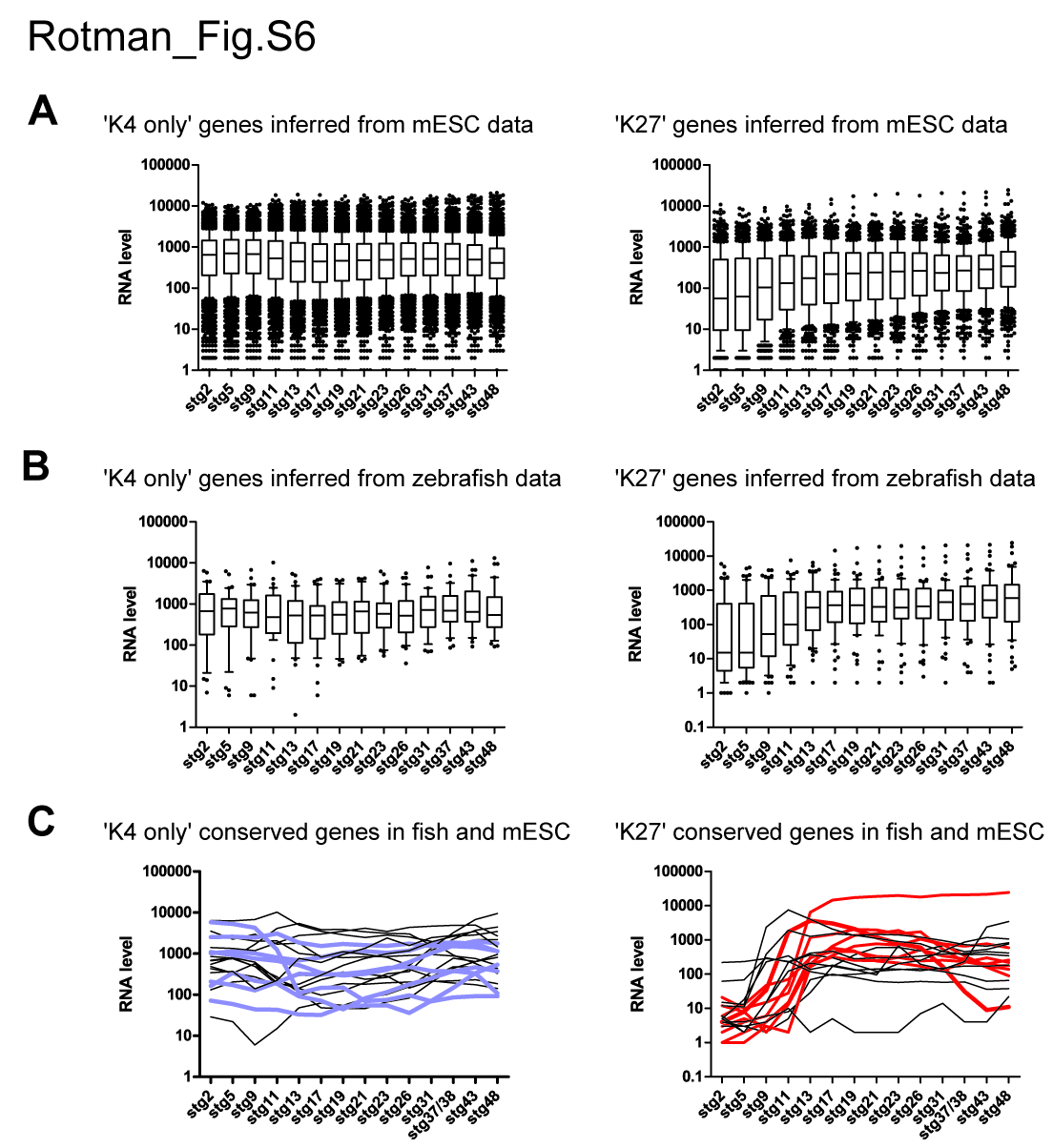

Supplement: Figure S6 — ‘K27’ and ‘K4 only’ genes have distinct kinetics of expression. The expression profile of ‘K27’ and ‘K4 only’ genes (see the main text for the description of the classes) is presented at different stages of X. laevis development using data from [23]. (A) Genes inferred from mouse ESC (mESC) data; (B) genes inferred from zebrafish data; (C) conserved ‘K4 only’ and ‘K27’ genes in zebrafish and mESC. Bold blue lines in c correspond to validated ‘K4 only’ genes while red lines are for validated ‘K27’ genes. In (A) and (B) the rectangles delineate the 25th and 75th percentiles, the horizontal bar is the median, and the whiskers indicate the 10th and 90th percentiles. (TIF) [file pone.0083300.s006.tif]

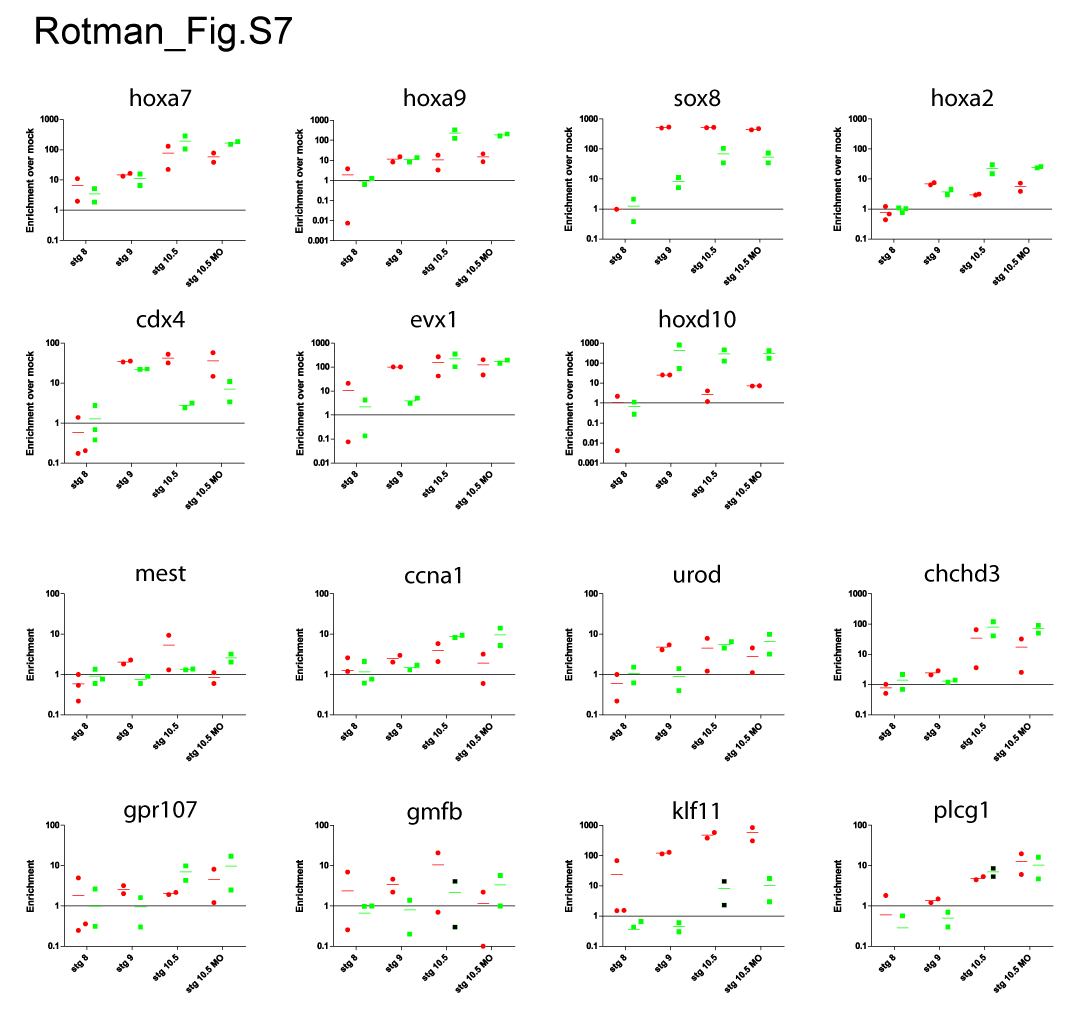

Supplement: Figure S7 — Individual data from ChIP experiments presented in Figure 5B . ChIP data obtained with the H3K4me3 antibody are presented in red and those obtained with the H3K27me3 antibody are in green. Each point represents an independent experiment. (TIF) [file pone.0083300.s007.tif]
