# Supplementary material for: PPARβ Interprets a Chromatin Signature of Pluripotency to Promote Embryonic Differentiation at Gastrulation
Source: PLoS One. 2013 Dec 18;8(12):e83300. doi: 10.1371/journal.pone.0083300 (PMC3867458; doi:10.1371/journal.pone.0083300)
Supplement: Protocol S1 — Functional analysis of RNA-seq data and Most-induced and most-decreased transcripts at gastrulation definition. (DOCX) [file pone.0083300.s010.docx]

**Protocol S1**

*Functional analysis of RNA-seq data*

We combined information from Xenbase ([ftp.xenbase.org](ftp://ftp.xenbase.org)) and Ensembl to assign when possible the corresponding mouse and/or human ortholog to each of the *X. laevis* transcripts. Starting with RefSeq accession numbers, we first added Unigene IDs using the ‘GenePageLaevisEntrezGeneUnigeneMapping.txt’ file available on Xenbase, which then allowed us to add the Xenbase gene name. We assigned a *Xenopus* *tropicalis* Ensembl accession number to each transcript using the ‘GenePageEnsemblModelMapping.txt’ provided on Xenbase. We then used the ensembl datamart to extract putative orthologs of *X. tropicalis* and mapped them by proxy onto the RefSeq *X.* *laevis* mRNAs. The lists of *X. laevis* and human orthologous genes with their relative expression data are given in Table S1.

We used BINGO [22] for the Gene Ontology term analysis on human genes and used resources from <http://www.broadinstitute.org/gsea/index.jsp> for the Gene Set Enrichment Analysis. The gene-permutation option was set to compute the enrichment scores.

To establish gene sets for the most-studied signalling pathways (Wnt, BMP, Nodal, FGF) and germ layer specification (endoderm, mesoderm, neuroectoderm) and for examples of early differentiation processes (somitogenesis and neural crest formation), we used the abundant bibliography on *X. laevis* development. We also used resources from published microarrays and annotation-type expression data ([ftp.xenbase.org](ftp://ftp.xenbase.org)) to build additional unbiased gene sets. Gene sets are given in Table S2.

*Most-induced and most-decreased transcripts at gastrulation*

Using data from [23], *X. laevis* transcripts were ranked according to the following gastrulation variation index (I):

I=(RNAlevel_stg13_)/RNAlevel_stg12_+RNAlevel_stg5_)/2

The 100 genes with the highest index and the 100 genes with the lowest index are considered in Figure 2D. In Figure 3A, *X. laevis* orthologs of mouse or zebrafish genes for which the H3K27me3 and H3K4me3 states were described at the pluripotent stage are ranked according to the above-mentioned index.
