# Supplementary material for: PPARβ Interprets a Chromatin Signature of Pluripotency to Promote Embryonic Differentiation at Gastrulation
Source: PLoS One. 2013 Dec 18;8(12):e83300. doi: 10.1371/journal.pone.0083300 (PMC3867458; doi:10.1371/journal.pone.0083300)
Supplement: Discussion S1 — Choice of epigenetic signatures of pluripotency. (DOCX) [file pone.0083300.s011.docx]

**Supplemental Discussion**

*Choice of epigenetic signatures of pluripotency*

To assign *X. laevis* genes a putative H3K4me3 and H3K27me3 state, we used ChIP-seq data from zebrafish [8], both because zebrafish is phylogenetically close to *X. laevis* and because the data were obtained from developing embryos. However, this dataset is of limited size, and we also used ChIP-seq data obtained from mouse orthologous genes that represented a much larger dataset [9]. We did not use data obtained from ChIP experiments from *X. tropicalis* with H3K4me3 and H3K27me3 antibodies [27] because they were obtained at mid-gastrula stage, which corresponds to the beginning of differentiation well after the pluripotent stage of the embryonic cells. In fact, our results for 15 genes, presented in Figure 3, clearly show that gastrula stage cells are not equivalent to earlier pluripotent cells in fish and mice, whereas late blastula cells are. Also, the authors [27] did not identify bivalent genes in *X. tropicalis* gastrula, whereas they were found in mouse ESCs and zebrafish blastula [8]. Therefore, as already discussed by others [8], we believe that data from *X. tropicalis* gastrulae do not represent a signature of pluripotency because gastrula cells are already engage in differentiation.
